# MOB2 Loss Sensitizes Lung Cancer Cells to PARP Inhibition Through p53-Dependent DNA Damage Signaling

**DOI:** 10.3390/cimb48030295

**Published:** 2026-03-10

**Authors:** Ramazan Gundogdu

**Affiliations:** 1 Department of Pharmacy Services, Vocational School of Health Services, Bingol University, 12000 Bingol, Türkiye; rgundogdu@bingol.edu.tr; 2 Department of Molecular Biology and Genetics, Institute of Science, Bingol University, 12000 Bingol, Türkiye

**Keywords:** PARP inhibitors, hMOB2, DNA damage response, lung cancer

## Abstract

Poly(ADP-ribose) polymerase (PARP) inhibitors exploit defects in homologous recombination (HR) but show limited and heterogeneous efficacy in non-small-cell lung cancer (NSCLC), where canonical HR deficiency is uncommon. Identifying alternative molecular determinants that modulate PARP inhibitor sensitivity therefore remains an important objective. In this study, we examined the role of the NDR/Hippo-associated cofactor human MOB2 (hMOB2) in shaping PARP inhibitor responses in lung cancer cells. hMOB2 was depleted by siRNA in A549 and H1299 cell lines, and cell viability, long-term survival, DNA damage, and apoptosis were assessed using WST-1 assays, clonogenic assays, Western blotting, immunofluorescence, comet assays, and caspase-3 activity assays. p53 dependency was evaluated using p53-null H1299 cells and p53 reconstitution via retroviral transduction. hMOB2 depletion sensitized A549 cells to olaparib and rucaparib, resulting in a marked reduction in long-term clonogenic survival. This effect was associated with enhanced p53 phosphorylation, persistent γH2AX accumulation, increased DNA strand breaks, and caspase-3-dependent apoptosis, while hMOB2 loss alone was not intrinsically cytotoxic. Sensitization required functional p53, as it was absent in p53-null cells but restored upon p53 re-expression. These findings suggest that hMOB2 contributes to PARP inhibitor responses in lung cancer cells and underscore the complexity of PARP inhibitor sensitivity beyond classical HR deficiency.

## 1. Introduction

Lung cancer remains the leading cause of cancer-related mortality worldwide [1], largely due to its aggressive biology, late diagnosis and high propensity for therapeutic resistance. Although targeted therapies—particularly tyrosine kinase inhibitors (TKIs)—and immunotherapies have significantly improved outcomes for selected patient subsets, durable responses remain limited by both intrinsic and acquired resistance mechanisms [2,3]. Beyond canonical resistance pathways driven by secondary mutations or bypass signaling, increasing evidence indicates that alterations in the DNA damage response (DDR) network contribute to therapeutic escape in non–small-cell lung cancer (NSCLC), including in TKI-resistant settings [4]. Dysregulated DDR signaling and enhanced DNA repair capacity have been implicated as adaptive mechanisms which sustain tumor survival under therapeutic pressure. These observations underscore the need to identify additional molecular vulnerabilities and rational combinatorial strategies capable of enhancing cytotoxic responses in lung cancer cells.

Poly(ADP-ribose) polymerase (PARP) enzymes are key regulators of genomic stability, primarily functioning in the detection and repair of DNA single-strand breaks through the base excision repair pathway [5,6]. Upon sensing DNA damage, PARP1 and PARP2 catalyze poly(ADP-ribosyl)ation of target proteins, facilitating the recruitment of DNA repair factors and coordinating chromatin remodeling at damage sites [6,7]. Pharmacological inhibition of PARP leads to the accumulation of unrepaired DNA lesions and the collapse of replication forks, ultimately converting single-strand breaks (SSBs) into cytotoxic double-strand breaks (DSBs), particularly in cells with defective homologous recombination (HR) repair [8,9]. This synthetic lethal principle has been successfully exploited in the clinic, resulting in the approval of several PARP inhibitors—such as olaparib, niraparib, rucaparib, and talazoparib—for the treatment of BRCA-mutant ovarian, breast, prostate and pancreatic cancers [10]. However, outside of classical HR-deficient contexts, the efficacy of PARP inhibitors remains limited by innate or acquired resistance [11,12,13], underscoring the importance of identifying additional molecular determinants which modulate PARP inhibitor sensitivity, especially in cancers such as lung cancer where HR defects are less prevalent [14]. Although PARP inhibitors hold context-dependent therapeutic potential in lung cancer [15], their efficacy is strongly influenced by the molecular landscape of DNA repair signaling [16,17]. This has driven growing interest in identifying novel genetic or signaling determinants that sensitize lung cancer cells to PARP inhibition, thereby expanding the applicability of this therapeutic strategy beyond canonical homologous recombination-deficient tumors [18].

In this context, increasing attention has turned toward non-canonical regulators of the DNA damage response (DDR) which may condition sensitivity to PARP inhibition in lung cancer. One such candidate is human MOB kinase activator 2 (hMOB2), a conserved adaptor protein classically associated with Hippo pathway signaling through its regulation of NDR/LATS kinases [19,20,21]. Beyond its established role in cell proliferation and survival, emerging evidence implicates hMOB2 in the cellular response to genotoxic stress, including the modulation of checkpoint control and genome stability [22,23]. Given that PARP inhibition exerts its cytotoxic effects by amplifying replication-associated DNA damage, alterations in hMOB2-dependent signaling may lower the threshold for PARP inhibitor-induced lethality [24]. This positions hMOB2 as a potentially important, yet underexplored, determinant of PARP inhibitor responsiveness in lung cancer cells.

In this study, we demonstrate that loss of hMOB2 markedly sensitizes A549 lung cancer cells to the PARP inhibitors olaparib and rucaparib, as evidenced by reduced cell viability and impaired long-term clonogenic survival. Mechanistically, PARP inhibition in hMOB2-deficient cells leads to enhanced DNA damage accumulation, as shown by increased p53 phosphorylation, sustained γH2AX foci formation and elevated DNA strand breaks detected by comet assay, culminating in apoptotic cell death assessed by caspase-3 activation. Importantly, this sensitization is dependent on intact p53 signaling, as hMOB2 inhibition failed to confer olaparib sensitivity in p53-deficient H1299 lung cancer cells, whereas ectopic restoration of p53 expression reinstated PARP inhibitor responsiveness. Together, these findings suggest hMOB2 as a potential determinant of PARP inhibitor sensitivity in lung cancer and reveal a critical role for p53 activation in mediating the cytotoxic effects of combined hMOB2 loss and PARP inhibition.

## 2. Materials and Methods

### 2.1. Cell Culture, Transfections and Cell Treatments

The human lung cancer cell lines A549 (ATCC, CCL-185, CVCL-0023) and H1299 (ATCC, CRL-5803, CVCL-0060) were kindly provided by Dr. Alexander Hergovich (Evotec, Toulouse, France) and maintained in Dulbecco’s Modified Eagle’s Medium (DMEM; Sigma-Aldrich, St. Louis, MO, USA) supplemented with 10% fetal calf serum (FCS; Gibco, Thermo Fisher Scientific, Waltham, MA, USA). Cells were routinely cultured under standard conditions and experiments were performed using exponentially growing cultures. Penicillin (100 U/mL) and streptomycin (100 µg/mL) were added to the culture medium as standard antimicrobial supplements. For gene silencing, cells were transfected with siRNAs (Qiagen, Hilden, Germany; siCTRL: 5′-aacgtacgcggaatacttcga-3′; siMOB2#4: 5′-caggagagacgtgtcagacga-3′) using Lipofectamine RNAiMAX (Invitrogen, Carlsbad, CA, USA) according to the manufacturer’s instructions.

### 2.2. Generation of Stable Cell Lines

Retroviral pools were generated using the pBABE-puro vector in PT67 retrovirus packaging cells as reported [22,24]. PT67 cells were transiently transfected with the respective plasmids using Lipofectamine 2000 (Invitrogen, Carlsbad, CA, USA) according to the manufacturer’s instructions. Viral supernatants were collected at 24 and 48 h post-transfection, clarified by filtration to remove cellular debris and used to infect target cells in the presence of polybrene. Infections were performed in two consecutive rounds to enhance transduction efficiency. Following transduction, cells were selected with puromycin (1.5 μg/mL) to establish stable polyclonal populations for downstream analyses. All cell culture and retroviral transduction procedures were conducted in accordance with Good Cell Culture Practice (GCCP) guidelines and institutional biosafety regulations. Cell lines were routinely tested for mycoplasma contamination using standard detection methods and were confirmed to be mycoplasma-free prior to experimental use.

### 2.3. WST-1 Cell Viability Assay

Cell viability and proliferation were assessed using the WST-1 assay, a colorimetric method based on cellular metabolic activity, performed according to the protocol described by Graham et al. [25]. Following transfection, cells were seeded into 96-well plates at a density of 5 × 10^3^ cells per well and incubated at 37 °C in a humidified atmosphere containing 5% CO_2_. Seventy-two hours after the corresponding treatments, 10 μL of WST-1 tetrazolium salt reagent (Roche Diagnostics GmbH, Mannheim, Germany) was added to each well and cells were further incubated for 4 h under the same conditions. Absorbance was measured at 450 nm with background correction at 650 nm using a microplate reader (Multiskan™ FC microplate reader, Thermo Fisher Scientific, Waltham, MA, USA). Data were analyzed after subtraction of background and blank control values and normalized to the corresponding untreated controls.

### 2.4. Western Blot Analysis

To detect protein expression levels, whole-cell lysates were resolved by 6% or 12% sodium dodecyl sulfate–polyacrylamide gel electrophoresis (SDS-PAGE) and transferred onto polyvinylidene difluoride (PVDF) membranes (Millipore, Billerica, MA, USA). Membranes were blocked in Tris-buffered saline containing 0.5% Tween-20 (TBST; 50 mM Tris-HCl, 150 mM NaCl, pH 7.5) supplemented with 5% (*w*/*v*) skim milk powder and incubated overnight at 4 °C with the indicated primary antibodies. For detection of proteins, including hMOB2 (home-made, [24]), p-p53 (S15) (Cell Signaling Technology, Danvers, MA, USA, #9284), p21 (Cell Signaling Technology, Danvers, MA, USA, #2947), tubulin (home-made), actin (Santa Cruz, Dallas, TX, USA, #sc-1616), GAPDH (Santa Cruz, Dallas, TX, USA, #sc-365062), membranes were blocked and incubated in TBST containing 5% bovine serum albumin (BSA) to minimize non-specific binding. Following washing, membranes were incubated with horseradish peroxidase-conjugated secondary antibodies (ThermoFisher, Waltham, MA, USA: anti-rabbit #31460, anti-mouse #31430, anti-rat #31470; SC/Insight Biotech, Wembley, UK: anti-goat #sc-2056) and immunoreactive bands were visualized using enhanced chemiluminescence (ECL; Amersham, GE Healthcare, Little Chalfont, UK) according to the manufacturer’s instructions. Densitometric analysis was performed using ImageJ (version 1.54, NIH, Bethesda, MD, USA) software, with protein levels normalized to the corresponding loading controls.

### 2.5. Immunofluorescence Analysis

Cells were processed for immunofluorescence as previously described [22,24]. Briefly, cells grown on glass coverslips were fixed in a solution containing 3% paraformaldehyde and 2% sucrose for 15 min at room temperature, permeabilized for 2 min with 0.5% (*v*/*v*) Triton X-100 in phosphate-buffered saline (PBS; Gibco, Waltham, MA, USA) and blocked in PBS containing 10% goat serum. Coverslips were then incubated overnight at 4 °C with the indicated primary antibodies. The following day, cells were incubated with the appropriate secondary antibodies and DAPI (Sigma-Aldrich, St. Louis, MO, USA) for 2 h at room temperature. Coverslips were mounted using Vectashield mounting medium (Vector Laboratories, Burlingame, CA, USA). Primary and secondary antibodies used for immunofluorescence were γH2AX (Cell Signaling Technology, Danvers, MA, USA, #9718; 1:50) and anti-rabbit Texas Red (Jackson ImmunoResearch via Stratech, Cambridge, UK, #711-075-152; 1:100). Images were acquired using an ApoTome fluorescence microscope (Zeiss, Oberkochen, Germany) equipped with a 40× objective and processed using AxioVision AxioVS40 software (v4.8.1.0; Zeiss, Oberkochen, Germany). For analysis, images were acquired using identical exposure settings across all experimental conditions. Foci were quantified manually in a blinded manner by counting γH2AX nuclear foci in at least 100 cells per condition from a minimum of three independent biological experiments. Cells exhibiting discrete nuclear γH2AX foci were scored and the number of foci per nucleus was calculated. Data are presented as mean ± SEM and statistical analyses were performed as indicated in the figure legends.

### 2.6. Single-Cell Gel Electrophoresis (Comet) Assay

Single-cell gel electrophoresis (comet) assays were performed as previously described [22]. Following electrophoresis, individual cells were visualized using an inverted microscope (Nikon, Tokyo, Japan) and analyzed using Komet Analysis software version 4.02 (Andor Technology Ltd., Belfast, UK). DNA damage was quantified using the Olive tail moment as the primary parameter. For each sample, time point and independent experiment, at least 100 cells were randomly selected and analyzed from duplicate slides. Image acquisition and comet scoring were performed in a blinded manner to eliminate observer bias. Quantitative data from individual cells were pooled and used for statistical analysis as described in the figure legends.

### 2.7. Caspase-3 Activity Assay

Caspase-3 activity was assessed using a Caspase-3 Colorimetric Assay Kit (R&D Systems, Minneapolis, MN, USA) according to the manufacturer’s instructions and the established literature protocols [26]. Briefly, cells (3 × 10^5^ per well) were seeded into 24-well plates, allowed to adhere overnight and treated with the indicated concentrations of olaparib or rucaparib for 72 h. Cells were then harvested, washed with cold phosphate-buffered saline (PBS; Gibco, Waltham, MA, USA) and lysed in the supplied lysis buffer on ice for 10 min. Lysates were clarified by centrifugation at 10,000× *g* for 1 min at 4 °C, and the resulting supernatants were collected. Equal amounts of protein from each sample were incubated with the caspase-3–specific colorimetric substrate (DEVD-pNA) in reaction buffer and transferred to a flat-bottom 96-well plate. Samples were incubated at 37 °C for 1 h and caspase-3 activity was quantified by measuring the release of p-nitroaniline at 405 nm using a microplate reader. Caspase-3 activity was normalized to total protein content and expressed relative to the corresponding untreated control. All experiments were performed using at least three independent biological replicates. Data are presented as mean ± SEM and statistical significance was assessed as described in the figure legends.

### 2.8. Clonogenic Survival Assay

Clonogenic assays were performed as previously described [25]. Briefly, cells were seeded at predetermined densities in 6-well plates or 6 cm culture dishes and allowed to adhere for 24 h prior to irradiation or drug treatment, as indicated, followed by three washes with fresh medium. Cells were subsequently maintained in complete growth medium, which was replaced every 3 days, until colonies reached a size of more than 50 cells. Colonies were then fixed in methanol/acetic acid (3:1) for 5 min and stained with 0.5% (*w*/*v*) crystal violet (Sigma-Aldrich, St. Louis, MO, USA) for 15 min. Colonies containing more than 50 cells were counted in a blinded manner to minimize observer bias. All clonogenic survival experiments were performed using at least three independent biological replicates. The surviving fraction was calculated by normalizing colony numbers to the plating efficiency of the corresponding untreated control cells, as previously described [27]. Quantitative data are presented as mean ± SEM and statistical analyses were conducted using appropriate tests as specified in the figure legends, with *p* < 0.05 considered statistically significant.

### 2.9. Graphics and Statistical Analysis

Graphical representation and statistical analyses were performed using GraphPad Prism software (version 8.0; GraphPad Software, San Diego, CA, USA). Data are presented as mean ± SEM, unless otherwise stated. For comparisons testing pre-specified, directional hypotheses—specifically assessing whether hMOB2 depletion increases sensitivity to PARP inhibition—a one-tailed, unpaired Student’s *t*-test was applied unless indicated otherwise. Statistical significance was defined as *p* < 0.05. Exact *p*-values and details of the statistical tests used are provided in the corresponding figure legends.

## 3. Results

### 3.1. hMOB2 Loss Sensitizes A549 Lung Cancer Cells to PARP Inhibition

**Figure 1:**
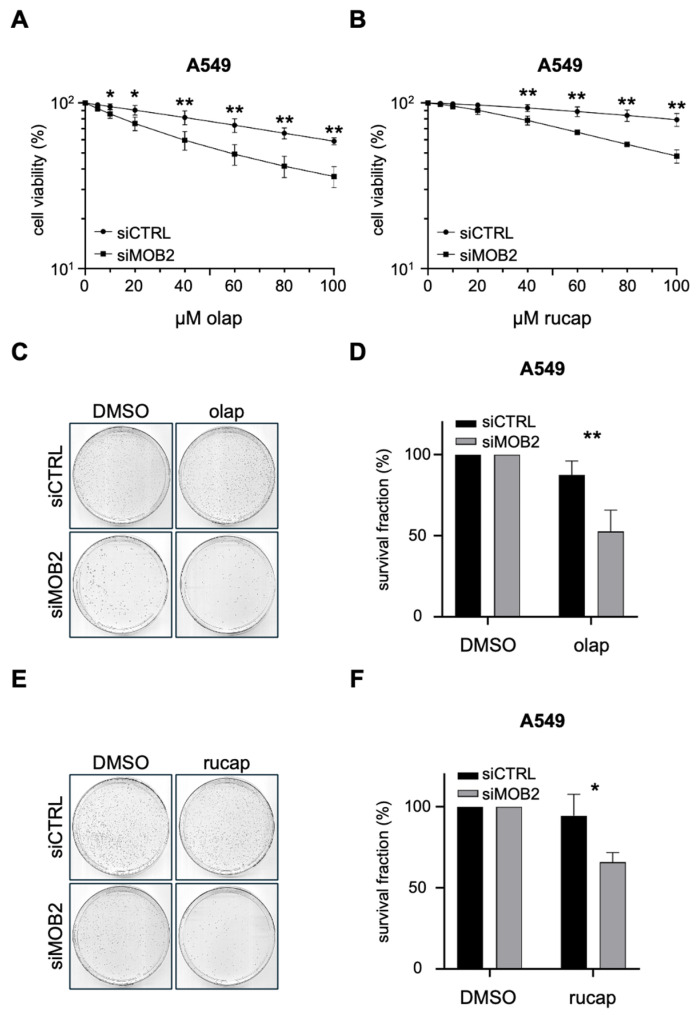
hMOB2 depletion sensitizes A549 lung cancer cells to PARP inhibition and impairs long-term clonogenic survival. (**A**,**B**) Short-term cell viability of A549 cells transfected with control (siCTRL) or hMOB2-targeting siRNA (siMOB2) and treated for 72 h with increasing concentrations (0–100 μM) of olaparib (**A**) or rucaparib (**B**), as assessed by WST-1 assay. Data are expressed relative to untreated controls and represent mean ± SEM from three independent experiments performed in triplicate. Statistical significance was assessed using one-way ANOVA followed by Dunnett’s post hoc test to compare each treatment condition with the corresponding control. The *p*-values for panel (**A**) are as follows: 10 μM: 0.02; 20 μM: 0.017; 40 μM: 0.005; 60 μM: 0.005; 80 μM: 0.002; 100 μM: 0.002. The *p*-values for panel (**B**) are as follows: 40 μM: 0.002; 60 μM: 0.002; 80 μM: 0.002; 100 μM: 0.002. (**C**,**D**) Representative clonogenic survival images (**C**) and quantification of surviving fraction (**D**) of A549 cells transfected with siCTRL or siMOB2 and treated with vehicle (DMSO) or olaparib (20 μM) for 8–12 days (2000 cells were seeded per dish). Surviving fraction was calculated relative to the corresponding untreated controls. The *p*-value for panel (**D**) is as follows olap: 0.007. (**E**,**F**) Representative clonogenic survival images (**E**) and quantification of surviving fraction (**F**) of A549 cells transfected with siCTRL or siMOB2 and treated with vehicle (DMSO) or rucaparib (20 μM) for 8–12 days. The *p*-value for panel (**F**) is as follows olap: 0.03. Clonogenic data represent mean ± SEM from at least three independent experiments, with colonies counted in a blinded manner. *p* < 0.05 (*), *p* < 0.01 (**). Olap: olaparib; Rucap: rucaparib.

To determine whether hMOB2 modulates the response of lung cancer cells to PARP inhibition, we performed 72 h WST-1 viability assays in A549 cells following siRNA-mediated depletion of hMOB2 and treatment with increasing doses of olaparib or rucaparib (0–100 μM). The 72 h time point was selected based on the pharmacodynamic properties of PARP inhibitors, which induce cytotoxicity primarily through replication-associated DNA damage [28]. This process requires at least one to two cell cycles to allow accumulation of unrepaired DNA lesions, replication fork collapse, and subsequent activation of apoptotic signaling pathways. Thus, a 72 h exposure window enables assessment of sustained DNA damage responses and their functional consequences on cell viability. In control-transfected A549 cells, both PARP inhibitors produced only a modest, concentration-dependent reduction in WST-1 signal (Figure 1A,B), consistent with the widely appreciated observation that many NSCLC models—including A549—exhibit limited intrinsic sensitivity to PARP inhibition in the absence of canonical HR deficiency [18,29]. Importantly, depletion of hMOB2 resulted in a clear leftward shift in the dose–response curves for both olaparib (Figure 1A) and rucaparib (Figure 1B), with substantially lower viability at matched drug concentrations compared with control cells, indicating that hMOB2 loss increases vulnerability to PARP inhibition.

To determine whether the enhanced PARP inhibitor sensitivity observed in short-term viability assays translated into a sustained loss of proliferative capacity, we next assessed long-term clonogenic survival in A549 lung cancer cells following hMOB2 depletion. As 72 h PARP inhibitor treatment was insufficient to fully capture the synergistic cytotoxic effects of hMOB2 loss in our preliminary experiments, cells were continuously exposed to the indicated concentrations of PARP inhibitors throughout the clonogenic assay period. Accordingly, control and hMOB2-silenced cells were treated with either vehicle (DMSO) or a fixed concentration (20 μM) of olaparib or rucaparib for 8–12 days, after which colony formation was evaluated. Under control conditions, A549 cells formed robust colonies irrespective of hMOB2 status, indicating that hMOB2 depletion alone does not substantially impair clonogenic growth (Figure 1C–F). Consistent with previous reports describing limited clonogenic sensitivity of NSCLC cells to PARP inhibition [29], treatment with olaparib or rucaparib produced only a partial reduction in colony formation in siCTRL cells. In striking contrast, hMOB2-depleted cells exhibited a pronounced loss of clonogenic survival following exposure to either PARP inhibitor, as evidenced by a marked reduction in both colony number and size (Figure 1C,E). Quantitative analysis confirmed a significant decrease in surviving fraction in siMOB2 cells compared with control cells treated with olaparib (Figure 1D; *p* < 0.01) or rucaparib (Figure 1F; *p* < 0.05). Collectively, these data demonstrate that hMOB2 loss converts PARP inhibition from a largely cytostatic or weakly cytotoxic insult into a potent suppressor of long-term clonogenic survival in A549 lung cancer cells.

### 3.2. hMOB2 Loss Converts PARP Inhibitor-Induced DNA Damage into Persistent Lesions and Apoptotic Cell Death

**Figure 2:**
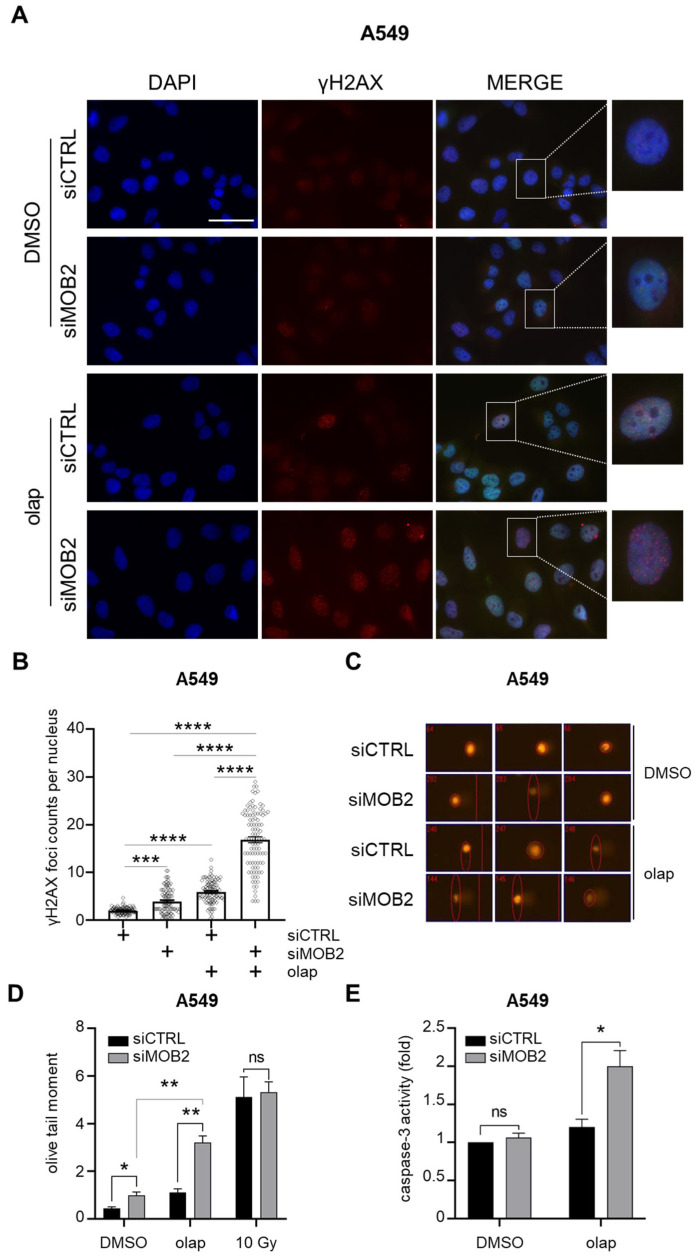
hMOB2 depletion promotes persistent DNA damage accumulation and apoptotic cell death following PARP inhibition. (**A**) Representative immunofluorescence images showing γH2AX (Ser139) foci formation in A549 cells transfected with control (siCTRL) or hMOB2-targeting siRNA (siMOB2) and treated with vehicle (DMSO) or olaparib (20 μM) for 72 h. Nuclei were counterstained with DAPI. (**B**) Quantification of γH2AX foci per nucleus from cells treated as in (**A**). Data represent mean ± SEM from three independent experiments, with at least 100 cells analyzed per condition in a blinded manner. Statistical significance was assessed using one-way ANOVA with Tukey’s multiple comparisons test. The *p*-values for panel (**B**) are as follows: siCTRL/DMSO-siMOB2/DMSO: 0.0003; siCTRL/DMSO-siCTRL/olap: 0.00003; siCTRL/DMSO-siMOB2/olap: 0.00002; siMOB2/DMSO-siMOB2/olap: 0.00002; siCTRL/olap-siMOB2/olap: 0.00007. (**C**) Representative images from single-cell gel electrophoresis (comet assay) performed in A549 cells transfected with siCTRL or siMOB2 and treated with olaparib (20 μM) for 72 h. Increased comet tail formation indicates elevated DNA strand breaks. (**D**) Quantification of DNA damage using Olive tail moment from comet assays performed as in (**C**). Data represent mean ± SEM from three independent experiments, with at least 100 cells scored per condition in a blinded manner. Statistical significance was assessed using unpaired Student’s *t*-test. The *p*-values for panel (**D**) are as follows: siCTRL/DMSO-siMOB2/DMSO: 0.02; siCTRL/olap-siMOB2/olap: 0.005; siMOB2/DMSO-siMOB2/olap: 0.002. (**E**) Caspase-3 activity in A549 cells transfected with siCTRL or siMOB2 and treated with olaparib (20 μM) for 72 h. Caspase-3 activity was measured using a colorimetric assay and normalized to untreated controls. Data represent mean ± SEM from three independent experiments performed in triplicate. Statistical significance was assessed using one-way ANOVA with Dunnett’s post hoc test. The *p*-value for panel (**E**) is as follows 0.04. *p* < 0.05 (*), *p* < 0.01 (**), *p* < 0.001 (***), *p* < 0.0001 (****). Magnification bar: 20 µm. ns: non-significant.

To determine whether the enhanced PARP inhibitor cytotoxicity observed upon hMOB2 depletion translated into persistent physical DNA lesions and apoptotic commitment, we combined spatial, single-cell and functional apoptosis assays. First, γH2AX foci formation was analyzed by immunofluorescence microscopy following 72 h treatment with olaparib (20 μM). Quantification of γH2AX foci revealed a significant increase in basal DNA damage signaling in hMOB2-depleted cells compared with control cells (Figure 2A,B), indicating that loss of hMOB2 alone modestly elevates endogenous DNA damage as expected [22]. Treatment of control cells with olaparib also resulted in a significant increase in γH2AX foci, consistent with PARP inhibition-induced replication-associated DNA damage (Figure 2A,B). Notably, olaparib treatment of hMOB2-depleted cells produced a further and highly significant increase in γH2AX foci compared with either control, hMOB2 depletion or olaparib treatment alone (Figure 2A,B). This additive elevation indicates that hMOB2 loss markedly intensifies PARP inhibitor-induced DNA damage accumulation. Collectively, these data demonstrate that hMOB2 deficiency lowers the threshold for γH2AX foci formation and promotes persistent DNA damage signaling in response to PARP inhibition.

To directly assess whether this sustained γH2AX signaling reflected increased DNA strand break accumulation, we next performed single-cell gel electrophoresis (comet) assays under identical treatment conditions. While control cells displayed minimal tail formation following olaparib exposure, hMOB2-deficient cells exhibited pronounced comet tails (Figure 2C), indicative of elevated DNA strand break burden [30]. Quantitative analysis using Olive tail moment confirmed a significant increase in DNA damage in hMOB2-depleted cells treated with olaparib compared with either condition alone (Figure 2D). Importantly, hMOB2 depletion in the absence of PARP inhibition caused only minimal changes in comet parameters, indicating that hMOB2 loss is not intrinsically genotoxic in A549 lung cancer cells but specifically intensifies DNA damage in the context of PARP inhibition.

Finally, to determine whether the persistence of DNA damage culminated in apoptotic cell death, caspase-3 activity was assessed following olaparib treatment. Consistent with the limited cytotoxicity of PARP inhibitors in many NSCLC models [18,29], control cells exhibited only a modest increase in caspase-3 activity (Figure 2E). In contrast, hMOB2-depleted cells showed a pronounced activation of caspase-3 upon olaparib treatment (Figure 2E), indicating robust induction of apoptosis. Notably, hMOB2 depletion alone did not significantly activate caspase-3, demonstrating that apoptosis is selectively triggered when hMOB2 loss is coupled with PARP inhibition.

Taken together, these data establish a mechanistic progression in which hMOB2 loss enhances PARP inhibitor-induced DNA damage beyond a repairable threshold, leading to persistent γH2AX-marked lesions, accumulation of DNA strand breaks and subsequent activation of the apoptotic execution machinery. By integrating spatial (γH2AX foci), physical (comet assay) and functional (caspase-3 activation) endpoints, these findings demonstrate that hMOB2 depletion converts PARP inhibition from a largely tolerable genotoxic stress into an irreversible, apoptosis-inducing insult in A549 lung cancer cells.

### 3.3. p53 Is Required for hMOB2-Dependent Sensitization to PARP Inhibition in Lung Cancer Cells

**Figure 3:**
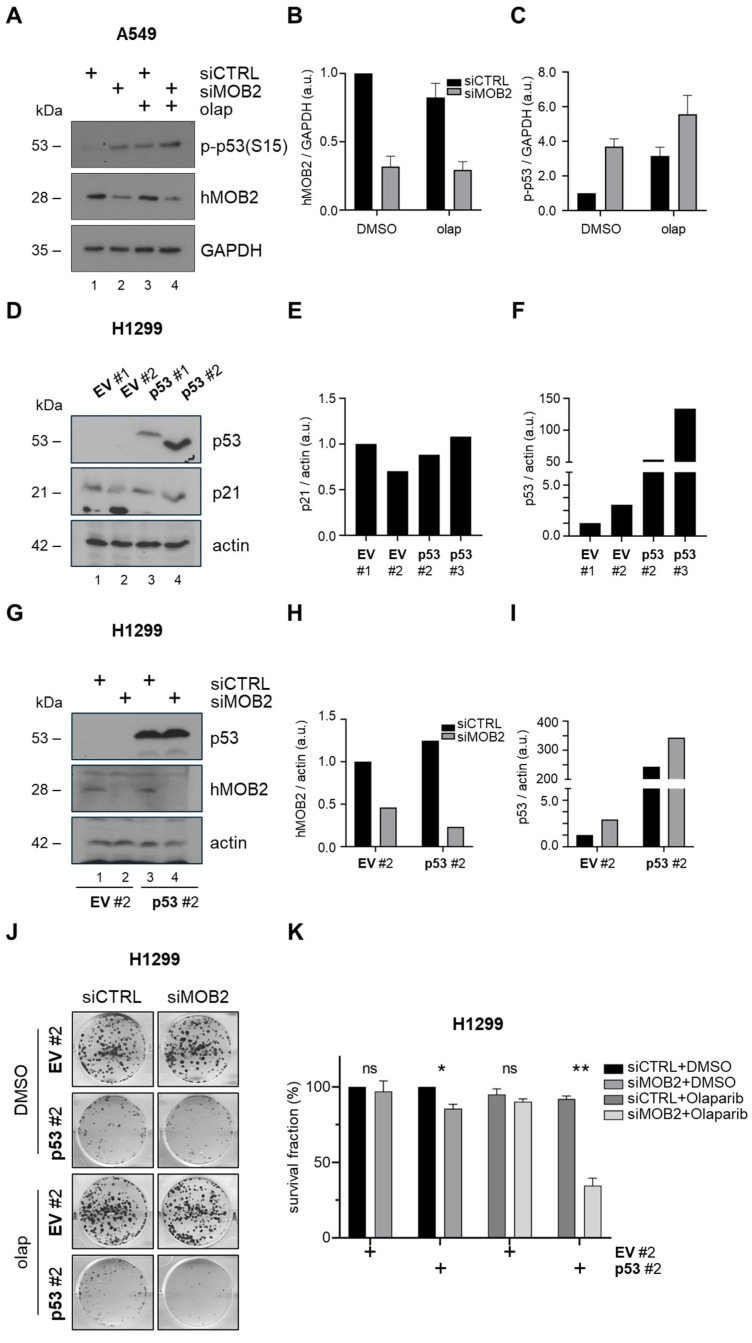
p53 is required for hMOB2-dependent sensitization to PARP inhibition in lung cancer cells. (**A**) Representative immunoblot analysis of phospho-*p53*, hMOB2 and loading control (*GAPDH*) in A549 cells transfected with control (siCTRL) or hMOB2-targeting siRNA (siMOB2) and treated with vehicle (DMSO) or olaparib (20 μM) for 72 h (*n* = 2). (**B**,**C**) Densitometric analysis of the immunoblot shown in Panel (**A**). (**D**) Immunoblot validation of stable p53 reconstitution in the p53-null H1299 lung cancer cell line following retroviral transduction with empty vector (EV) or wild-type p53-expressing constructs. Two independent p53-expressing populations are shown. Actin serves as a loading control (*n* = 1). (**E**,**F**) Densitometric analysis of the immunoblot shown in Panel (**D**). (**G**) Immunoblot confirmation of hMOB2 knockdown in p53-null or -reconstituted H1299 cells (replicate #2) following transfection with control (siCTRL) or hMOB2-targeting siRNA (siMOB2). Actin serves as a loading control (*n* = 1). (**H**,**I**) Densitometric analysis of the immunoblot shown in Panel (**G**). (**J**) Representative clonogenic survival images of p53-null or -reconstituted H1299 cells transfected with siCTRL or siMOB2 and treated with vehicle (DMSO) or olaparib (20 μM) for 8–12 days (1000 cells were seeded per well). (**K**) Quantification of clonogenic survival shown in (**C**), expressed as surviving fraction relative to untreated controls. Data represent mean ± SEM from at least three independent experiments. Statistical significance was assessed using unpaired Student’s *t*-test. *p* < 0.05 (*), *p* < 0.01 (**). *ns*: non-significant.

To mechanistically explain the pronounced reduction in long-term clonogenic survival observed upon combined hMOB2 depletion and PARP inhibition, we next examined activation of the DDR. Since PARP inhibition can convert unrepaired single-strand lesions into replication-associated DNA breaks and sustained checkpoint engagement [28,31]—particularly when genome maintenance pathways are compromised—we focused on p53 phosphorylation at the S15 residue, as central DDR readout. The level of p53 phosphorylation was minimal in control conditions and, as expected, weakly induced by either PARP or hMOB2 inhibition alone, whereas the strongest induction was observed upon combined hMOB2 and PARP inhibition (Figure 3A–C). To directly test whether the enhanced sensitivity to PARP inhibition observed upon hMOB2 loss is dependent on p53, we utilized the p53-null lung cancer cell line H1299. H1299 cells were transduced with a retroviral construct encoding wild-type p53 or the corresponding empty vector control to generate p53-reconstituted derivatives. Western blot analysis confirmed robust p53 protein expression in two independent p53-expressing H1299 populations (Figure 3D–F), whereas vector-transduced cells remained p53-negative. Notably, p21 protein levels were detectable in both control and p53-reconstituted cells and did not show a consistent increase upon p53 expression, consistent with previous reports indicating that basal p21 expression can be p53-independent and that p53-mediated p21 induction is context- and activation-dependent rather than constitutive [32,33,34]. In addition, the occasional appearance of a closely migrating secondary band is most likely attributable to post-translational modifications of p21, such as phosphorylation, which are known to alter its electrophoretic mobility [32,33]. Based on stable p53 expression, the second p53-reconstituted H1299 population was selected for downstream analyses. Efficient depletion of hMOB2 in these cells was first verified by Western blotting, confirming robust knockdown prior to functional assays (Figure 3G–I). We then examined the impact of hMOB2 loss on long-term clonogenic survival following PARP inhibition. In p53-reconstituted H1299 cells, inhibition of hMOB2 alone resulted in only a modest reduction in clonogenic growth, while treatment with olaparib alone did not significantly impair colony formation, indicating that neither perturbation is intrinsically cytotoxic in this setting (Figure 3J–K). In striking contrast, combined hMOB2 depletion and olaparib treatment led to a pronounced reduction in clonogenic survival, as evidenced by a marked decrease in both colony number and size (Figure 3J–K). Importantly, this sensitization phenotype was not observed in parental p53-null H1299 cells, in which hMOB2 depletion failed to enhance sensitivity to PARP inhibition (Figure 3J–K). Together, these findings demonstrate that restoration of p53 expression is sufficient to reinstate hMOB2-dependent sensitivity to PARP inhibition, establishing p53 as a critical downstream effector of this synthetic vulnerability. When integrated with the γH2AX accumulation, persistent DNA damage and apoptosis observed in p53-proficient A549 cells; these data support a model in which hMOB2 loss amplifies PARP inhibitor-induced DNA damage signaling which requires functional p53 to drive irreversible loss of clonogenic survival.

## 4. Discussion

Non-small-cell lung cancer (NSCLC) accounts for over 80% of all lung cancer cases and remains the principal contributor to lung cancer-related mortality worldwide [1]. Despite advances in screening and early detection, approximately two-thirds of patients are diagnosed with advanced or metastatic disease (stage IIIB–IV), for which systemic therapy—including cytotoxic chemotherapy, immune checkpoint inhibition, and molecularly targeted agents—constitutes the mainstay of treatment [35,36]. While these approaches have yielded meaningful improvements in response rates, overall survival and treatment tolerability in molecularly defined subsets of patients, durable clinical benefit remains limited for a substantial proportion of individuals. Consequently, there is an ongoing need to identify additional therapeutic vulnerabilities in NSCLC, a pursuit increasingly enabled by the expanding molecular and functional characterization of lung cancer biology [37]. PARP inhibitors have established a paradigm of synthetic lethality in HR-deficient cancers, most notably those harboring *BRCA1* or *BRCA2* mutations [8,9]. However, the extension of this therapeutic strategy to lung cancer has been limited by the relatively low prevalence of classical HR defects in NSCLC and by the frequent emergence of intrinsic resistance [17,29,38]. This has prompted considerable interest in identifying alternative molecular determinants which sensitize lung cancer cells to PARP inhibition. In this study, we identify hMOB2 as a potential modulator of PARP inhibitor sensitivity in lung cancer and define a mechanistic axis linking hMOB2 loss to amplified DNA damage signaling and p53-dependent apoptosis. Our findings align with and extend prior work implicating hMOB2 in genome maintenance [19,20,22,24]. hMOB2 has been shown to act as a negative regulator of NDR1/2 kinases [19,20,39] and has been implicated in the regulation of DNA damage response signaling and genome maintenance pathways [22], including processes linked to HR [24]. Notably, previous work demonstrated that hMOB2 depletion leads to spontaneous DNA damage signaling, engagement of the ATM–CHK2 axis and a p53/p21-dependent G1/S checkpoint, supporting a role for hMOB2 in buffering endogenous genotoxic stress [22]. However, the relevance of this pathway in lung cancer—where HR deficiency is not a dominant feature—has remained unexplored. Our data demonstrate that hMOB2 loss is sufficient to create a PARP inhibitor-sensitive state in lung cancer cells, thereby broadening the functional relevance of hMOB2 beyond classical HR-deficient contexts.

A key aspect of our study is the emphasis on long-term clonogenic survival rather than short-term metabolic readouts alone. While WST-1 assays revealed enhanced sensitivity to olaparib and rucaparib upon hMOB2 depletion, the most striking effect was observed in clonogenic assays, which represent the gold standard for assessing irreversible loss of reproductive capacity following genotoxic stress [27]. This distinction is particularly important in NSCLC, where PARP inhibitors often induce cytostatic effects without durable cytotoxicity. Our results demonstrate that hMOB2 loss converts PARP inhibition from a largely tolerable insult into one that compromises long-term cell survival. Mechanistically, we show that hMOB2 depletion amplifies DNA damage signaling induced by PARP inhibition, as evidenced by increased p53 phosphorylation, sustained γH2AX foci formation and elevated DNA strand breaks detected by comet assay. This signaling pattern is consistent with unresolved replication-associated DNA lesions, which are known to arise when PARP-mediated repair is inhibited during S phase [13,31,38]. Importantly, hMOB2 loss alone did not induce extensive DNA damage, indicating that hMOB2 functions as a damage-buffering factor whose loss becomes critical only when PARP-dependent repair pathways are blocked. This is conceptually consistent with emerging models in which synthetic lethal interactions reflect the collapse of compensatory genome maintenance pathways rather than the presence of a single dominant repair defect [40,41]. In this context, our findings extend previous evidence that hMOB2 supports HR-associated processes, including RAD51 activation and nucleofilament formation, by demonstrating that hMOB2 loss exacerbates PARP inhibitor-induced replication stress and promotes persistent DSB signaling in lung cancer cells [24].

A central and novel finding of our study is the requirement for functional p53 in mediating hMOB2-dependent PARP inhibitor sensitivity. Activation of ATM and phosphorylation of p53 are canonical responses to persistent DNA damage and replication stress [42,43]. In p53-proficient A549 cells, enhanced DNA damage signaling following hMOB2 loss culminated in caspase-3 activation and apoptotic cell death. In contrast, p53-null H1299 cells failed to undergo PARP inhibitor sensitization upon hMOB2 depletion, despite effective knockdown and PARP inhibition. Crucially, restoration of p53 expression in H1299 cells reinstated hMOB2-dependent sensitivity, establishing p53 as a necessary downstream effector rather than a correlative marker. This requirement for p53 is consistent with earlier observations that hMOB2 loss engages canonical DDR checkpoint outputs [22] and suggests that the cytotoxic consequence of combined hMOB2 and PARP perturbation is shaped not only by lesion burden but also by tumor-suppressor pathway integrity.

Early-phase studies in NSCLC and small-cell lung cancer have indicated biological activity of PARP inhibitors—particularly in combination regimens—yet clinical responses remain heterogeneous [16,18,37]. In this context, our data suggest that loss or functional attenuation of hMOB2, together with intact p53 signaling, may define a subset of lung cancers with heightened vulnerability to PARP inhibition; notably, *TP53* remains wild type in a clinically relevant fraction of NSCLC, making this axis potentially actionable in selected patients [44]. Nevertheless, the variability observed in the clinic is unlikely to be explained by any single biomarker, including hMOB2 status alone, as PARP inhibitor response is influenced by a complex interplay of the broader DDR landscape, baseline replication stress, drug-specific PARP trapping potency, checkpoint adaptation and acquired resistance mechanisms which restore repair capacity or stabilize stalled replication forks [18,28,38,45]. Accordingly, we propose that hMOB2 should be viewed as one component of a composite vulnerability—most plausibly within *TP53*-proficient tumors—rather than a universal determinant of clinical response. Consistent with prior work suggesting that MOB2 expression may correlate with PARP inhibitor sensitivity in selected cellular contexts [24], future integrative analyses will be required to determine whether hMOB2 contributes to broader vulnerability signatures in lung cancer rather than serving as a standalone predictive biomarker. Future work should integrate hMOB2 expression with HRD/replication-stress signatures and DDR gene alterations in large patient cohorts (e.g., TCGA) and validate predictive value in patient-derived models, to establish translational relevance and to identify molecular subsets of lung cancer most likely to benefit from PARP inhibition. Several limitations warrant consideration. Our study was conducted in established cell line models, and validation in additional cell lines with various genetic backgrounds, patient-derived models and in vivo systems will be necessary to assess clinical relevance. In addition, the functional role of hMOB2 was interrogated using a single siRNA sequence, and future validation using an independent siRNA or rescue experiments with an siRNA-resistant hMOB2 construct will be necessary to definitively confirm specificity and formally exclude potential off-target effects. Furthermore, while our data clearly implicate p53 signaling downstream of hMOB2 loss, the precise molecular mechanisms by which hMOB2 regulates DNA repair or replication stress responses remain to be elucidated. Dissecting whether hMOB2 directly influences HR factor recruitment, replication fork stability or checkpoint adaptation in lung cancer cells will be an important focus of future work.

## 5. Conclusions

This study suggests hMOB2 as a potential modulator of PARP inhibitor sensitivity in lung cancer and establishes a mechanistic framework linking hMOB2 loss to amplified DNA damage signaling and p53-dependent apoptotic execution. These findings expand the conceptual basis for PARP inhibitor responsiveness beyond classical HR deficiency and reveal a previously unrecognized therapeutic vulnerability. If validated in future studies with translational models, the hMOB2–p53 axis may enable rational patient stratification and expand the therapeutic utility of PARP inhibition in lung cancer.

## Data Availability

The original contributions presented in this study are included in the article. Further inquiries can be directed to the corresponding author.

## Abbreviations

The following abbreviations are used in this manuscript:

ATM	Ataxia telangiectasia mutated
BRCA	Breast cancer susceptibility gene
DDR	DNA damage response
DMSO	Dimethyl sulfoxide
DSB	DNA double-strand break
H2AX	H2A histone family member X
HR	Homologous recombination
hMOB2	Human Mps One Binder protein
NDR	Nuclear Dbf2-related kinase
NSCLC	Non–small-cell lung cancer
PARP	Poly(ADP-ribose) polymerase
siRNA	small interfering RNA
SSB	DNA single-strand break
